# Liver Transplantation for Alcoholic Liver Disease and Hepatocellular Carcinoma

**DOI:** 10.3390/cancers10020046

**Published:** 2018-02-09

**Authors:** Patrizia Burra, Alberto Zanetto, Giacomo Germani

**Affiliations:** Multivisceral Transplant Unit, Department of Surgery, Oncology and Gastroenterology, Padua University Hospital, Via Giustiniani 2, 35128 Padua, Italy; alberto.zanetto@yahoo.it (A.Z.); germani.giacomo@gmail.com (G.G.)

**Keywords:** cirrhosis, alcohol, hepatocellular carcinoma, carcinogenesis, surveillance, liver transplantation

## Abstract

Hepatocellular carcinoma is one of the main important causes of cancer-related death and its mortality is increasingly worldwide. In Europe, alcohol abuse accounts for approximately half of all liver cancer cases and it will become the leading cause of hepatocellular carcinoma in the next future with the sharp decline of chronic viral hepatitis. The pathophysiology of alcohol-induced carcinogenesis involves acetaldehyde catabolism, oxidative stress and chronic liver inflammation. Genetic background plays also a significant role and specific patterns of gene mutations in alcohol-related hepatocellular carcinoma have been characterized. Survival is higher in patients who undergo specific surveillance programmes than in patients who do not. However, patients with alcohol cirrhosis present a significantly greater risk of liver decompensation than those with cirrhosis due to other aetiologies. Furthermore, the adherence to screening program can be suboptimal. Liver transplant for patients with Milan-in hepatocellular carcinoma represents the best possible treatment in case of tumour recurrence/progression despite loco-regional or surgical treatments. Long-term result after liver transplantation for alcohol related liver disease is good. However, cardiovascular disease and de novo malignancies can significantly hamper patients’ survival and should be carefully considered by transplant team. In this review, we have focused on the evolution of alcohol-related hepatocellular carcinoma epidemiology and risk factors as well as on liver transplantation in alcoholic patients with and without hepatocellular carcinoma.

## 1. Introduction

Hepatocellular carcinoma (HCC), arising in patients with liver cirrhosis, is the second most lethal cancer worldwide, with persistently increasing mortality in Europe, North/South America and Africa [1,2,3,4].

Liver cirrhosis and HCC are the major life-limiting consequences of progressive fibrotic liver diseases, mainly caused by chronic viral infection, alcohol excessive consumption and non-alcoholic fatty liver disease [5].

Despite chronic hepatitis B still represents the main cause of liver cancers worldwide [6], the burden of alcohol-related HCC is generally increasing [7]. Nowadays, in Europe, alcohol excessive consumption accounts for 40–50% of all liver cancer cases [8] and, with the sharp decline of chronic viral hepatitis, it has been estimated that alcohol will become the leading cause of liver cancer [7,9,10], at least in developed countries. With this regard, the “Global Burden of Disease Liver Cancer Collaboration Group” recently estimated that one third of liver cancers deaths are related to alcohol abuse [2].

In the present review, we will highlight the most relevant data about current epidemiology and risk factors, alcohol-related hepatic carcinogenesis, surveillance strategies of alcohol-related HCC. In the second section of the manuscript, we will focus on liver transplantation, emphasizing the indication and management strategies, the general pre-transplant evaluation of alcoholic patients and long-term outcome of transplanted patients.

## 2. Alcohol Consumption and HCC: Epidemiology and Risk Factors

Alcohol excessive consumption is common in the Americas and Western Europe. Furthermore, Alcohol consumption is rising in Asia too [11].

Approximately 7% of the adults in the United States meets the criteria for alcohol abuse/dependence [12]. In Italy, more than one third of hospitalized patients presented a clinical history characterized by excessive alcohol consumption (more than 60 g of alcohol per day) [13]. In the last fifty years, alcohol intake has doubled in United Kingdom [14]. Similarly, in Asia, alcohol consumption has risen dramatically, especially in men [15] and in young people, as a negative effect of the globalisation [16].

Several studies have focused on the relationship between alcohol abuse and HCC development. In a study including 1605 HCC patients, the hospitalization rates for HCC in alcoholic patients were more than 70% greater than the ones in patients with chronic hepatitis C [17]. 

The odds ratio (HR) for HCC in patients who reported alcohol use was 2.4 (95% CI: 1.3–4.4) when compared with non-drinkers in a recent case-control study including 115 patients [18]. In those drinking less than 80 g of alcohol per day, the adjusted HR was not significantly increased. On the contrary, in those who were drinking more than 80 g daily turned out to be significantly higher (HR: 4.5).

In a recent meta-analysis, including 19 prospective studies, a positive association between the amount of alcohol intake and the risk of HCC was found. In particular, patients who drank three alcoholic units per day had a 16% increased risk of HCC, those who drank six units per day had a 22% increased risk [19].

The relationship between ethanol consumption and HCC risk was prospectively evaluated in a Taiwanese study [20]. More than ten thousand men were followed-up for of nine years. One hundred and twelve HCC were diagnosed during 110.039 years of cumulative follow-up. The authors found a relative risk for HCC of 1.6 (95% CI: 1.0–2.6) in non-infected patients with active alcohol consumption in comparison with non-infected and non-drinkers patients. Unfortunately, the alcohol intake was not well characterized and defined either as “non-drinking” or “drinking” only.

To summarize, in those countries where there was a significant alcohol consumption and a moderate prevalence of viral hepatitis (i.e., Italy and United States), as well as in those countries with a greater prevalence of viral hepatitis but a lower prevalence of alcohol use (i.e., Japan), chronic alcohol use is related with a 2-fold increased HR for development of HCC. When the alcohol consumption is heavy, the HR rises up to 5–7-fold increase.

Once liver cirrhosis is established, the risk of HCC development significantly rises [21,22,23]. Among patients with compensated disease, 2–4% of them develop HCC yearly [24,25]. Conversely, in cohort studies of non-cirrhotic alcoholic patients, the summary HCC incidence rates are lower (0.01 per 100 person-years) [21,23,26,27,28,29,30].

In a recent study by Marot et al. [31], the relevance of the underlying aetiology of liver disease on both HCC occurrence and death risk was evaluated in a group of 752 patients (70% of them with alcoholic cirrhosis, 19% with HCV cirrhosis and 11% with metabolic cirrhosis). The cumulative incidence rate of HCC was lower in patients with alcohol related liver disease than in those with HCV and fatty liver (8.4% vs. 22.0% vs. 23.7% after 10 years; *p* < 0.001). Although alcoholic patients had a reduced risk of HCC development (0.39; 95% CI, 0.20–0.76; *p* = 0.005), they presented a greater risk of death (1.53; 95% CI, 1.20–1.95; *p* < 0.001), due to a higher decompensation rate of baseline liver disease.

In a similar way, Bucci et al. [32] investigated the impact of alcohol aetiology on presentation, treatment and outcome of HCC in cirrhotic patients, comparing a group of alcoholic patients with a group of patients with HCV-related disease. A group of 1642 HCV and 573 alcoholic patients were compared for different biochemical, clinical and outcome variables. Patients with alcohol related liver disease were younger, male and presented an HCC diagnosed outside surveillance, more frequently in intermediate/terminal stage. Furthermore, they had a baseline worse liver function. Interestingly, after adjustment for the lead-time, the median overall survival was lower in alcoholic patients than in those with HCV infection (27.4 months vs. 33.6 months; *p* = 0.02) reflecting that alcohol hampered survival through its effects on secondary prevention as well as on cancer presentation. However, it was not associated with a greater cancer aggressiveness or worse treatment result. 

West et al. [33] estimated the risk of developing HCC according to the baseline aetiology in a population-based study in the United Kingdom. Among 3107 cirrhotic patients, the adjusted relative risk of HCC was increased between twofold and threefold amongst patients with viral and autoimmune/metabolic aetiologies, compared to those with alcohol-associated cirrhosis. Ten-year predicted cumulative incidence estimates of HCC for alcohol was 1.2%, compared with 4% of patients with chronic viral hepatitis (*p* < 0.05).

To sum up, alcoholic patients seem to present a lower risk of developing HCC than patients with viral hepatitis or non-alcoholic fatty liver disease. However, when HCC is diagnosed, prognosis is worse than other aetiologies.

The magnitude of HCC risk reduction following abstinence has not been yet established due to limited scientific evidence. A meta-analysis of four cohort studies showed that abstinence reduced the risk of developing HCC by 6–7% annually in cirrhotic patients, despite a large uncertainty in the estimate and more than two decades required to normalize the risk to the level of never drinkers [34,35]. 

In the study by Donato et al. [36], the HR for HCC development was higher (5) in patients who stopped alcohol use within the past five years than in current drinkers. Only after more than ten years of abstinence, HR returned to the baseline risk value for current drinkers. This paradox may be justified by the fact that patients stopped alcohol consumption when they become decompensated. Therefore, the discontinuation of drinking within the previous five years could be only a surrogate of the end stage liver disease status. Secondly, patients who quit drinking may live longer, being therefore exposed to a greater risk for HCC.

If alcohol should be considered a carcinogen still has to be clearly confirmed. However, it was found that heavy alcohol consumption was a significant risk factor for HCC development in non-cirrhotic patients. In a pathological study, 19% of patients with non-cirrhotic alcoholic liver disease developed HCC [37]. Similarly, in an Italian single-centre cohort including 174 patients with a first diagnosis of HCC, 142 of them had underlying liver cirrhosis. Among the HCC cases in whom liver cirrhosis was excluded (21), a histological picture of alcoholic hepatitis was found in 5 (24%) patients [38]. Probably, in these patients, a favourable genetic background plays a role in HCC development too [39].

## 3. Alcohol-Related Hepatic Carcinogenesis

The pathways of alcohol-induced liver carcinogenesis are very complex [40]. Up to date, several synergistic pathways have been described [41] and a detailed analysis goes beyond the scope of the present manuscript. The main important ones involve acetaldehyde catabolism, oxidative stress with peroxidation of the membrane lipids as well as chronic inflammation injury.

The liver metabolizes ethanol to acetaldehyde by alcohol dehydrogenase and cytochrome P450 2E1 (CYP2E1). Acetaldehyde “per se” presents direct and indirect carcinogenic actions that have been characterized in vitro and in vivo (i.e., DNA damage and alteration of protein structure and function [42], impairment of DNA repair mechanisms [43], mitochondria damage [44].

In patients with significant chronic alcohol consumption, a 10–20 fold increase of CYP2E1 activity has been characterized. The increase in CYP2E1 function lead to a burst in reactive oxygen species, including peroxides, superoxide, hydroxyl radical, and singlet oxygen, that leads to lipid peroxidation and enhances oxidative cellular stress [45]. The boosted production of reactive oxygen species is associated with a depletion of glutathione, which is one of the main important intracellular antioxidants. Moreover, it has been associated with a significant mitochondrial dysfunction [46].

In patients with chronic alcohol use, the intestinal iron absorption as well as the hepatic iron stores are inappropriately increased, being the iron overload a well recognized risk factor for HCC [47]. From a molecular point of view, the presence of intracellular free iron can be associated with an increased generation of ROS, through the Fenton reaction or through lipid peroxidation [48]. 

“Cancer immune-surveillance” is the process by which an organism’s immune system recognized transformed cells in order to inhibit the growth of neoplastic tissue [49]. In this setting, Natural Killer (NK) cells play a major role, especially in the “elimination” phase. In cirrhotic patients, several alterations regarding NK cell number and lytic function have been described [50], particularly in those with alcohol related disease [51]. More specifically, in patients with alcoholic cirrhosis and active alcohol consumption, an increased number of NK circulating cells have been described. However, the cells were characterized by a significant reduction of normal physiological lytic capacity [50]. 

In patients with chronic consumption of alcohol, the function of other organs, such as the gut, is impaired too. [52]. Ethanol alters the quantitative and qualitative composition of the microbiota, but also hampers the constitutional barrier function of intestinal epithelial, leading to an increased release of bacteria and bacterial products that “fuel” the inflammatory response in the liver [53]. The release of lipopolysaccharide into the splanchnic circulation is associated with an increased release of pro-inflammatory mediators (i.e., Interleukin 6) by Kupffer cells, via toll-like receptors [54,55]. Elevated level of IL-6 interferes with DNA repair therefore promoting the neoplastic process. Moreover, it up-regulates MCL-1, which is an onco-suppressor gene [56].

## 4. Surveillance

In cirrhotic patients, the annual incidence of HCC justifies periodic 6-monthly ultrasound screening. The aim of screening programme is to diagnose the tumour as early as possible, when it can be treated with a curative intent. Indeed, different studies confirmed that patients who are under regular HCC surveillance have greater survival rates than patients who are not [57,58].

With regard to alcoholic aetiology, no specific recommendations can be made regarding a specific schedule of screening. Alcoholic cirrhotic patients should therefore be followed-up according to the general guidelines for patients with liver cirrhosis [22].

Cirrhotic patients are very heterogeneous and present with a different oncological risk, according to the presence of one or more known risk factors. Given that, the principal limit of screening program is the fact it is not personalized according to specific patient risk. Ideally, if clinical and/or molecular risk scores were able to capture the “single-patients” risk of developing HCC, they would enable a rational allocation of the resources to the “high-risk” patients who most need the intervention avoid at the same time ineffective and wasteful distribution of the demanding screening efforts to “low-risk” individuals [5].

In alcoholic patients, classical HCC risk factors include clinical variables such as age and presence of metabolic syndrome stigmata as well as the severity of underlying liver disease [25]. The integration of such risk factors may be helpful in selecting those who may benefit of different surveillance schemes.

In the study by Mancebo et al. [24], data regarding the surveillance in 450 alcoholic cirrhotic patients (Child Pugh A or B), aged 40 to 75 years, were evaluated. Over the follow-up period, 62 patients developed HCC with an annual incidence of 2.6%. The risk of HCC was independently associated at the multivariate analysis to the following parameters: age ≥55 years (HR, 2.39; 95% CI, 1.27–4.51) and platelet counts less than 125 × 10^3^/mm^3^ (HR, 3.29; 95% CI, 1.39–7.85). Taken togheter, these variables were combined to set 3 different categories at risk. Among patients without risk factors, the annual incidence of HCC was 0.3% (*n* = 93), if 1 risk factor was present the annual incidence increased up to 2.6% (*n* = 228) whereas in patients with two risk factors it was 4.8% (*n* = 129) (*p* < 0.0001).

Obesity has become an important risk factor for non-liver neoplasms. Indeed, malignancies represent the second most important cause of death among patients with fatty liver disease [59]. With regard to HCC, N’Kontchou G. et al. [25] prospectively evaluated the impact of overweight on the risk of HCC development among 478 well-compensated cirrhotic patients and showed the presence of a positive linear relationship between the increase of body mass index and HCC risk. More particularly, BMI between 25–30 kg/m^2^ was associated with HR of 2.0 (95% CI, 1.4–2.7) while BMI of 30 kg/m^2^ or more was associated with HR of 2.8 (95% CI, 2.0–4.0).

Lastly, in the paper by Nahon and Nault [39] the genomic analysis of alcohol-related HCC showed the presence of somatic mutations in some typical genes such as ARID1A, CTNNB1, SMARCA2 and TERT. These genes modulate the HCC pathways (i.e., ethanol/lipid/iron metabolism, metabolism or oxidative stress) previously described. Together with the classical risk factor above mentioned, the combination of such genetic background with epidemiological and clinical data might better define specific HCC risk classes in order to best adapt screening strategies. 

Clinicians should face also patients’ adherence, which is crucial for the success of a screening program. Active alcohol consumption has been described as a strong predictor (HR, 3.03; 95% CI, 2.03–4.51) of suboptimal adherence in a recent study by Mancebo et al. [60]. These patients have a more advanced HCC stage at diagnosis and, unfortunately, tend to be less frequently treated with curative intention.

## 5. Liver Transplantation for Hepatocellular Carcinoma: Indications for Listing and Patient Management in the Waiting List

The outcome of the early series of patients transplanted for hepatocellular carcinoma, with broad selection criteria, was very poor in terms of survival (five-year survival <40%) due to the high risk of recurrence (between 32 and 54% at five years) [61,62]. This made HCC a contraindication for liver transplantation (LT) until 1996, when the Milan criteria (MC) (1 lesion ≤5 cm, 3 lesions with no one >3 cm, no vascular invasion and no metastasis) were introduced [63]. Overall, in patients transplanted within Milan criteria, 5-year survival rate (65–78%) was very similar to patients who were transplanted with non-HCC indications, according to both European and American studies (65–87%) [64].

Nowadays, LT represents the first treatment choice for patients with small multi-nodular HCC (≤ 3 nodules ≤ 3 cm) or those with single tumours ≤5 cm and advanced liver dysfunction [22]. In this patients population, LT can cure the tumour and the underlying cirrhosis, which predisposes to further hepatic carcinogenesis, at the same time. Whit this regard, there is no difference in the indications for listing as well as in the patient management during the waiting list period between HCC patients with alcohol related liver disease and HCC patients arising in the context of other aetiologies.

In previous studies it was showed that a slight extension of the MC limits could expand the number of potential liver transplant candidates without a significant decrease of survival [65]. Of note, the extension of HCC listing criteria must consider two different aspects at the same time: single patient condition on one hand and wait list pressure on the other. The transplant community established that any expansion of the MC must guarantee a five year post-transplant survival greater than 60% [66]. With this purpose, the transplant benefit, which may be achieved in HCC patients beyond MC, must be regulated to adequate levels of post-transplant utility. This is a crucial point in order to avoid any negative effect to the priority of patients waiting for LT for decompensated liver disease and not for HCC.

In the recent years, several Authors tried to identify the best possible extension of MC [67]. The University of San Francisco (UCSF) criteria (single tumour <6.5 cm, or up to 3 nodules <4.5 cm and a total sum <8 cm) [68] and the Up-to-Seven criteria (HCC with seven as the sum of size of the largest nodule and the number of nodules) [69] are characterized by a slight extension of Milan proposal. 

These two proposals, as well as Milan original propose, are based only on morphological evaluation of the HCC. Of note, all these criteria are unavoidably linked to the limits of the radiological evaluation and, notably, do not take into account other factors that have been related to patient survival such as the cancer biology as well as the response to treatment. To surmount these limitations, new listing criteria including alpha-fetoprotein (AFP) levels have been studied. In the study by Duvoux et al. [70], including 537 patients, patients who had serum AFP <100 ng/mL, even if exceeding MC from a morphological point of view, presented a similar risk of post transplant recurrence when compared to patients within MC. On the same line, the model proposed by Toso et al. [71] consider two different parameters: the first, morphological, total tumour volume (≤115 cm^3^) and the second, biological, AFP level (≤400 ng/mL). This model (“TTV/AFP”) was tested against MC to evaluate the drop out rate during the waiting list period and 4-years post transplant survival in a relatively small group of 38 patients with HCC beyond MC. Although the drop out rate was greater in case of HCC beyond Milan but within TTV/AFP model in comparison to those within MC (42.1% vs. 25.1%), there were no differences regarding HCC recurrence rate (9.4% vs. 4.5%) and post-transplant survival (74.6% vs. 78.7%).

In the oncological field, great effort has been made in order to identify a biological signature able to predict cancers biology. More particularly, in case of HCC, researches focused on phenotype influencing its progression and treatment response. Unfortunately, HCC is per se a heterogeneous tumour, being therefore very difficult to find reproducible molecular or genetic marker that could support clinicians in the clinical management of these patients [72].

From the clinical point of view, it would be more feasible to classify the tumour aggressiveness according to tumour stage and response to previous treatments. This concept has been recently proposed for the selection of HCC patients beyond MC in Italy [73]. This approach is based on the fact that patients who presented an HCC beyond MC but who achieved a good and continued response to treatments presented a similar post transplant outcome when compared to patients within MC [65]. With this in mind, a modern approach to evaluate the possibility of enlarging MC should be done with the following subsequent steps of patient global evaluation. Firstly, patient should be considered as liver transplant candidate only in case a 5 years post-LT survival of 60% can be guarantee (i.e., patients with a “transplantable tumour” (TT)). With this regard, any of the aforementioned criteria can be apply to select recipients with TT. At a later stage, selected patients whit a TT should be subsequently divided according to other “dynamic” factors reflecting tumour biology and therefore the need of early transplantation, including the response to bridging therapies, the achievement of down-staging within MC and the time of recurrence after treatment [74]. This approach can stratify patients with a TT in different subclasses with different prognosis, from patients with HCC completely treated and patients with a late recurrence (>2 years from a previous curative treatment) on one side, and patients with early recurrence or incomplete response to treatment who presented within conventional MC at the time of recurrence or after partial treatment response, on the opposite. Following this stratification, the prioritization for transplant can be calculated considering both the urgency principle (risk of drop out) and the utility principle (maximum survival post-LT). This dynamic process, which consider the treatment response as the most important selection criteria, is aimed to reduce the priority of being transplanted in those patients who can still wait, favouring those ones who, even if still with a transplantable tumour, present an increased risk of drop out following tumour growth. Theoretically speaking, this innovative approach could be useful to select HCC patients with the best possible transplant benefit, including also patients who present an HCC beyond MC. Finally, although original and clinically applicable, the expansion of MC must consider each transplant centre condition, including the risk of mortality during the waiting time. 

Strategies for the HCC management during the wait-list time (“bridging therapies”) may be significantly different according to different factors that reflect patients’ geographic location (i.e., anticipated waiting time) as well as by other factors such as centre expertise. With this regard, there is no difference between patients with alcohol and non alcohol-related HCC. Kulik et al. [75] recently reported a comprehensive analysis about the available literature (from 1996 to 2016) regarding the impact of bridging therapies (i.e., trans-catheter arterial chemoembolization, trans-arterial radio-embolization, ablation and radiotherapy) in the management of HCC patients awaiting LT. Both comparative and non-comparative studies were included whereas no randomized controlled trials were identified. Interestingly, independently from HCC type 1, 2 or 3, quality of evidence was very low due to high risk of bias, imprecision and inconsistency. In general (and particularly for HCC type 3), in HCC patients listed for LT, the use of bridging therapies is associated with a non-significant trend toward improved waitlist and post-transplant outcomes, though there is a high risk of selection bias in the available evidence. 

For those patients with an expected waiting time more than 6 months, bridging therapy can be used to prevent tumour progression and drop-out [65]. The type of therapy depends on several factors such as HCC size and location. Centre expertise should also be taken into account. As previously mentioned, the response to bridging therapy may be useful to select those HCC patients with a more aggressive behaviour [76]. How much time is needed to adequately define tumour biology? It is not known. Mehta et al. proposed that the minimum of 6 months is needed to reduce the risk of post-transplant recurrence [77]. On the same line, the lack of response to locoregional treatment (i.e., trans-arterial chemoembolization) has been related to a greater risk of dropout during the waiting list time as well as to a higher probability of tumour recurrence after liver transplantation, independently from the tumour burden [78,79]. The risk of HCC recurrence has been extensively evaluated. An inappropriate selection indeed has been associated with a decrease in overall survival, in particular among patients who underwent transplantation very early after the diagnosis [80]. If transplantation is performed too early after an oncological treatment, there may be no sufficient time for the biological behaviour of the tumour to become clear, favouring an increase in HCC recurrence rate. Given these considerations, UNOS (“United Network for Organ Sharing”) has recently modified the allocation policy for HCC patients, including in the algorithm also an observation period of 6-months. As marker of tumour biology, the decrease of AFP after treatments has been related to post-transplant outcome. More particularly, the AFP level closest to time of LT has reported to be an independent predictive factor of overall survival after LT [81,82] and in patients in whom a rise in AFP occurred, a significant increase in post transplant mortality has been described. Based on that, AFP “dynamic” level should be considered in the decision-making algorithm for the access to LT.

## 6. Pre-Transplant Evaluation, Waiting List Period Monitoring and Risk of Post-Transplant Relapse

In the recent years, the excellent outcome of patients transplanted for alcoholic liver disease have encouraged physicians to refer these patients to tertiary care hospital [83]. However, only a minor proportion of these patients meet the required criteria for LT [84]. Pre-transplant work-up is not different from that of patients with other aetiologies. However, these patients can present several comorbidities (i.e., cirrhotic cardiomyopathy, chronic pancreatitis, Wernicke’s encephalopathy, dementia, peripheral neuropathy, poor nutritional status and upper aero-digestive malignancies) that must be carefully ruled out [85,86].

One of the main challenges for the transplant team is the management of both clinical and psychosocial aspects. In this setting, a multidisciplinary evaluation is therefore needed, including psychiatric, psychological and addiction specialist evaluation. With regard to the psychiatric evaluation, it seems often advisable given the well know association between alcohol abuse and personality disorders, depression and other psychiatric disorders, that can also influence the post-transplant outcome of these patients [87].

The likelihood of long-term abstinence after transplantation should also be evaluated. With this regard, a 6-month period of abstinence (“six month rule”) before considering a candidate suitable for LT is still required in the majority of centres [88]. This rule has a dual objective: firstly, to avoid liver transplantation in those patients in who’s clinical condition will ameliorate, secondly, to select patients at greater risk of relapse. Nowadays, however, the applicability of this rule as predictor of relapse is still significantly controversial [85]. Indeed, the patient’s psycho-social evaluation seems more important for the identification of risk factors for alcohol relapse and thereafter for the establishment of the real probability of long-term abstinence. With this regard, patients admitted for acute alcoholic hepatitis are not considered eligible for LT in most transplant centres, though early transplantation, if performed in very selected patients after the first episode of severe decompensation with no response to steroid therapy, has been shown to improve survival [89]. In the study by Mathurin et al., LT has been proposed as rescue therapy in a very selected group of 26 patients with acute alcoholic hepatitis (AAH) (biopsy proven, first episode of decompensation) with no response to corticosteroids, after a strict selection process based on a multidisciplinary evaluation. Short (six months) and medium term (24 months) survival after LT were significantly higher than in matched controls (77% vs. 23%). Similarly, Im et al. confirmed the good results of early LT in a US group of 94 patients with AAH [90]. The rigid pre-transplant evaluation, in which the psychosocial evaluation represent the first “next-step” after the patient was considered a non responder to corticosteroid therapy, was associated with a significant reduction in term of access to LT (9.6% of patients), however, the six months survival was significantly higher than matched controls (89% vs. 11%). The most significant issue in these patients is the risk of relapse when liver transplant is performed in patients with active drinking. In the landmark paper already mentioned [89], about 10% of patients presented a relapse up to more than three years after LT. This could be relevant not only from the “single-patient” point of view, but also for the potential negative impact on donation rate. However, it seems from a recent multicentre survey that early transplantation for AAH would not negatively impact in terms of organ donation [91]. Patients must undergo a well-conducted and multidisciplinary selection with no evidence of clinically significant comorbidities. Furthermore, the social background, including the presence of an active supportive by the family, is mandatory. Then, clinicians should maintain a transparent as well as direct communication with the society, based on the concept of no “a priori” exclusion to the evaluation for LT in case of acute alcoholic hepatitis. In this clinical setting, further studies are needed for the identification of accepted selection criteria that can provide the best long-term results [92]. The LT option should therefore be explored within strict criteria in this setting, as indicated in a recent Italian position statement [93].

After inclusion in the waiting list, patients can resume alcohol consumption. Alcohol relapse rate may be underestimated if just self-reported by patients (or their relatives), particularly if they understand that this may represent an obstacle for being active in the waiting list. In cirrhotic patients, the typical serological markers of alcohol excessive consumption such as increase in gamma-glutamyl transpeptidase (GGT), the amino-transferase (AST)/alanine aminotransferase (ALT) ratio and the increased mean corpuscular volume (MCV) cannot be used given their low predictive value. The quantification of carbohydrate-deficient transferrin (CDT) has been proposed for the detection of continuous and significant alcohol consumption (50–80 g of alcohol per day) over a period of at least seven days [94]. In patients with moderate to heavy ethanol consumption, the carbohydrate content of transferrin synthesized by the hepatocytes decreases and, if combined with the increase of plasmatic level of GGT, its performance in detecting alcohol abuse present a relatively good sensitivity (90% and 75% in males and females, respectively) [95]. Unfortunately, in patients with hyperbilirubinemia the sensitivity of CDT significantly decrease. Furthermore, it cannot be used to identify the use of small amount of alcohol [96].

With this regard, other “short-term”, direct markers such as serum (sEtOH) and urinary ethanol (uEtOH) have been proposed in order to detect small quantity of alcohol (<10 g). The main limitation of both markers is the fact that their application is temporarily limited, due to the short time of alcohol detection (8 h in case of sEtOH and 24 h in case of uEtOH) [97,98]. Even with these limitations, Staufer et al. has demonstrated that uEtG can be used for the detection of alcohol relapse in transplant candidates as well as in transplanted patients, being the most relevant and independent predictor of consumption (OR 1.204; *p* < 0.001) [99].

The hair content of EtG represents a different toxicogical approach. Indeed, the metabolite persists in hairs for at least one month after ethanol consumption. Different cut-off have been proposed. Even if they are not still completely standardized, a cut-off of 7 pg/mg is a strong indicator of regular alcohol consumption, whereas a cut off level of 30 pg/mg can accurately detect heavy drinking patients [11].

After transplantation, patients may resume alcohol consumption. Should this be considered alcohol “relapse”? On one hand, alcohol relapse has been characterized as “any alcohol consumption occurring after transplantation”, whereas, on the other, as “drinking pattern associated with liver injury” only [100,101]. Given the absence of a well-standardized definition, different studies reported a wide range of alcohol relapse rates, ranging from 10% to 50% [102,103,104]. Another issue favouring this huge range is the different duration of follow-up after transplantation, which has been reported to be between six months and five years in different cohorts.

In the cohort described by Di Martini et al. [105], almost half of the patients referred alcohol use after LT. In 26% of the cases it was only “rare slips” whereas 19% of the liver transplant recipient reported, “harmful drinking”. Among the latter, in 30% of the cases the relapse occurred very soon after transplantation (within the first 24–36 months after LT) with a subsequent decrease over time, in 30% of the cases harmful drinking was reported to be continuative with no significant interruption after transplantation, and in the remaining a more late relapse was documented (at least three years post-LT). These results would suggest that no clear “time cut-off” can be made and therefore a close clinical monitoring is advisable also in the long term after LT.

Is the pre-transplant abstinence a useful toll for the prediction of alcohol relapse? In a recent systematic review including 22 studies [106], in only two of them the six months period of alcohol abstinence was found to be predictive of post-LT relapse. Importantly, other factors were investigated and described as more important predictors of relapse such as social background, presence of psychiatric disease, addiction to other substances, previous failed attempts at alcohol rehabilitation, younger age at transplantation and family history of alcoholism.

In the study by Yates et al. [107] a new score, the “High risk alcoholism relapse score (HRAR)”, was proposed with the aim of assessing the utility (and therefore the clinical applicability) of the six-month rule. This score has been calculated on the basis of the following variables: duration of alcohol abuse, number of daily drinks and number of previous alcoholism treatments. The Authors then compared the accuracy of HRAR score with the classic 6-month rule, showing that it was much better in predicting post-transplant relapse, with 79%, 69%, and 54% agreement between HRAR and six month abstinence for low, moderate and high HRAR groups respectively.

From a clinical point of view, which is the effect of alcohol relapse on graft function? Indeed, in transplant recipients who reported an occasional heavy drinking no significant alteration of graft function has been described [108], On the other hand, in another cohorts, patients with harmful drinking may present a lower long-term survival when compared with those totally abstinent or with recipients with minor relapse [109]. With regard to short-term outcome, the impact of harmful drinking seems to be very similar in patients transplanted for alcoholic liver disease and in patients transplanted for non-alcoholic liver diseases with no statistical difference in term of survival. However, in studies with a longer follow-up (five to ten years post-LT), survival rates significantly decreased (45–68% vs. 75–86%) [110,111,112]. Graft loss secondary to alcohol relapse seems to be an infrequent event, however, it has been described in patients who resumed harmful alcohol consumption very soon after LT. More specifically, Di Martini et al. showed that graft loss occurred more frequently in those patients with early relapse in comparison with those who were abstinent, had rare slips, or who experienced a late relapse of harmful ethanol intake (72%, 45%, 45% and 0%, respectively) [113]. Finally, in a recent series, [114] one third of the patients with a severe and prolonged alcohol relapse (41 out of 128, 32%) developed cirrhosis recurrence, which was associated with a reduced survival with respect to patients without severe relapse (49.7% and 21.0%, vs. 74.4% and 59.4% 10 and 15 years post-transplantation, respectively). 

## 7. Survival after Liver Transplantation for Alcohol Related Cirrhosis

Different studies confirmed that the long-term survival in patients who underwent liver transplantation for alcoholic liver disease is comparable with those of patients who were transplanted for other etiologies. In the landmark study based on European Liver Transplant Registry) database [115], evaluating patients transplanted between 1988–2005 in Europe, survival rates at 1, 3, 5 and 10 years after LT were 84%, 78%, 73% and 58%, respectively, significantly greater than those observed in patients transplanted for viral (HCV and HBV) cirrhosis (82%, 74%, 70%, 60%; *p* = 0.04) and in patients with cryptogenic cirrhosis (78%, 73%, 69%, 61%; *p* = 0.05). When survival outcome was evaluated according to HCV or HBV co-etiology, survival rates 1, 3, 5 and 10 years after LT were significantly lower in patients with HCV infection in comparison to patients with HBV infection (84%, 75%, 65%, 52% vs. 89%, 85%, 81%, 64%, respectively; *p* = 0.0002). 

However, after the recent introduction of direct acting antivirals, that have changed the landscape of HCV treatment also in the post-transplant setting, this will probably change [116]. In the study by Burra et al. [115], patients with HCC were excluded from the analysis and, to date, no other study has specifically investigated the outcome of this sub-group of patients. 

Cardiovascular disease and de novo malignancies represent the major causes of death in patients who underwent liver transplantation for alcohol related liver disease [117]. Briefly, these patients present a risk of de novo malignancies 1.5–2 folds higher than patients transplanted for other etiologies [118,119]. Furthermore, if the analysis is limited to upper aero-digestive neoplasms, the risk rises up to 10 fold [101]. Lastly, social aspect should not be underestimated in this patient population. Indeed, the amount of deaths attributable to social causes has been described to be higher in patients with alcoholic cirrhosis than in those with HBV/HCV infection or cryptogenic liver disease (1.3% vs. 0.7% vs. 0.4%, respectively; *p* = 0.03) [115]. 

## 8. Conclusions 

In conclusion, alcoholic excessive consumption represents a significant hamper for public health. Alcoholic liver disease includes a large spectrum of clinical manifestations, starting from fatty liver up to cirrhosis and hepatocellular carcinoma. Among patients with hepatocellular carcinoma, those with recurrent tumour after loco-regional or surgical treatment or the ones with cancer progression not responding to radiological or surgical treatment (or with progression of underlying cirrhosis) should be evaluated for liver transplantation. The indication to liver transplantation for alcoholic liver disease can be reported on up to 20% of cases in most of European and American liver transplant centres and alcoholic liver disease has become a well recognized indication for liver transplantation. Moreover, in the recent years both short and long-term graft and patient survival increased. However, patients with alcoholic cirrhosis (including those with hepatocellular carcinoma) should undergo a specific evaluation related to abuse of alcohol and alcohol abuse-related problems before listing, while in the waiting list and after transplantation giver the risk of relapse into alcohol consumption.
